# Water-Drinking Indications and Contra-Indications

**Published:** 1905-03-15

**Authors:** 


### WATER-DRINKING INDICATIONS AND CONTRA-INDICATIONS.

It is well known that artificial, dry heat causes evaporation of moisture from the binding of books and from joints of furniture, etc. That it should also cause increased surface evaporation in human beings is not surprising. Hence the necessity for drinking water at intervals during the day, especially for those in whom a certain amount of constipation with a tendency to hard stools shows that there is a diminished amount of water in the system, and that nature is only with difficulty able to carry on certain of her functions requiring water.

As the result of the successful employment of water to relieve many ordinary symptoms, it has become the custom to recommend water very generally and sometimes without sufficient deliberation. For the comparatively well, who need only to supply an equivalent for the moisture that is being lost—particularly through the skin and the lungs—the simple advice to drink plentifully of water is almost sure to be good. In many diseases, moreover, the ingestion of water is an efficient aid to other treatment. For example, in bronchitis, especially if the accompanying cough be hard and ineffective, the taking of large amounts of fluid is usually productive of good results. In fact, it is on this therapeutic principle that the reputation acquired by many of the old home remedies depends. A warm drink taken just before going to bed will often keep a patient from being annoyed by a cough during the night.

On the other hand, to recommend abundant drinking of water may be, for patients suffering with some diseases, decidedly poor advice. For example, in the case of cardiac affections with failing compensation, the necessity for an already weakened heart to overcome the resistance of a large amount of fluid in the circulation is certain to invite further deterioration of the heart muscle. One of the well-known and very serious forms of heart disease is the degenerative type which occurs in connection with the drinking of large quantities of such liquors as beer; the result is degeneration of the heart muscle and an extensive dilatation of the heart on account of overwork. It is easy to understand that a large amount of even so innocuous a fluid as water would tend to produce similar results. Whenever, then, there is loss of compensation in a heart that is laboring under some valvular defect, or under some degenerative process, the advice to drink plenty of water should be given with caution.

There is one class of cases, however, in which the unfortunately common custom of recommending the liberal drinking of water is likely to prove even more harmful than in heart disease. The first recommendation that too many physicians make to a patient suffering from almost any form of nephritis, unless there is marked ascites present, is to drink water abundantly. The idea is that in this way the system will be thoroughly "flushed out" and that the toxins in the circulation which may injure the kidneys will be diluted and made less harmful. The exclusive milk diet so often suggested for nephritis is really one expression of this old tradition. It must be remembered, however, that the value of this old-time advice lacks confirmation. Prof. von Noorden, in his monograph on nephritis, has particularly insisted on the fact that this advice is often fruitful of more harm than good. In the American edition of his work on nephritis he says that water is always very badly excreted by the kidneys when they are acutely diseased, and frequently also in subacute and subchronic forms of nephritis, though individual cases differ greatly in this respect. He insists that the only possible way to determine whether water, which is in many cases one of the best diuretics we have, is indicated or not, is to determine whether the output of water bears a definite relation to the amount ingested. He adds: "A condition of hydremia must be regarded as a constant source of irritation of the kidneys, and the problem that confronts us is to prevent excessive stimulation of these organs as much as possible."

It is evident, therefore, that water-drinking, usually a physiologic, a hygienic and a remedial procedure of great value, in special cases may be attended with harm, and that the physician's advice to individuals should be given with reference to the particular case under consideration.—*Jour. A. M. A.*

